# Miscellaneous

**Published:** 1890-02

**Authors:** 


### MISCELLANEOUS.

MOST of us eat too much and sleep to little; we read to much and thin k to little; we work too much and enjoy too little.

YOUNG DOCTOR: "They don't bleed people nowadays as they did twenty years ago, do they, Professor?" Professor: "Not *with the lancet.*"

WISTAR'S BALSAM OF WILD CHERRY—Is said to be mainly composed of wild cherry, ipecac, squills, opium, tartar emetic, syrup, alcohol, anise and water.

OWEN in his last hours, when on his dying bed, dictated a short letter to a friend. The amanuensis had written, "I am yet in the land of the living," when Owen at once arrested him: "Stop, alter that; write, I am yet in the land of the dying, but I hope soon to be in the land of the living."

"I GUESS dad wishes we'd all die and go to heaven," said a miser's son to his maternal parent. "Why so?" she asked, upon recovering from her astonishment. "Oh, 'cause heaven's such a cheap place to live in."

CLOTHS dipped in very hot water, wrung out and instantly applied to the seat of pain, will be frequently of very great service. In some instances it adds to the efficacy to employ substances having medical properties as well. In every process of fomentation there should be two flannels, each three yards long, with the ends sewed together, and one flannel should be got ready while the other is on.

DANDRUFF.—Dandruff is frequently a catarrh of the scalp. It is an abnormal exfoliation of the scales from the skin of the head. Dead cells or scales are constantly exfoliated from every portion of the body. The scalp should be thoroughly brushed and rubbed, but it is best not to use a comb that scratches. A good dry brushing of the scalp every day is the best prevention. The only suitable hair-brush is one of bristles—not wire—arranged in little tufts, irregular in length.

RHEUMATIC GOUT.—Rheumatic gout is an incurable disease. It is a sort of degeneration of the cartilages of the joints. The change is organic, and nothing can be done for it. But when it is due to lowered nerve tone, can be improved and the system raised from its low state of vitality by proper nutrition, the disease can be arrested. But patients of this class are never improved by reducing treatment, and it is a mistake to rush such off to various springs for the purpose of taking hot baths and similar treatment.

A NEW DRUG FOR USE IN DIABETES.—Of great value, has recently appeared in the market. It consists of powdered jambul seeds—the seed of a plant, *Syzygium jambolanum* or *Eugenia jambolana,* found in various parts of India, the Mauritius, Ceylon and the United States of Columbia. It has been well tested by the medical faculty in England, Germany and the United States, and it is said to be a promising remedy in all cases of diabetes. The action of the drug is to prevent formation of sugar in the system, and so to stay waste; and cases are on record showing that under its influence the special restrictive diet so obnoxious to diabetes patients can be dispensed with.

### NEW PUBLICATIONS.

THE DIXIE DOCTOR, Atlanta, Ga., T. H. Huzza, M. D., editor. $1.50 a year.

BELFORD'S MAGAZINE begins the New Year with a feast of good things suited to every taste. We welcome it to our sanctum.

MEDICAL MIRROR, a new medical Journal, makes its first appearance with the New Year in very commendable shape. St. Louis. I. N. Love, editor.

THE TRAINED NURSE. The holiday number of this excellent monthly is a model of neatness and good taste, besides being the forerunner of the New York Trained Nurses Association.

VICKS' FLORAL GUIDE for 1890, is simply gorgeous! It is a profusely illustrated pamphlet of 100—8x10 in.—pages. Price, 10 cents. Lovers of flowers will be well repaid by sending for it to James Vick, seedsman, Rochester, N. Y.

THE CONTURY. "Bubostis," by Amelia B. Edwards, with its many illustrations in the January Number, is a complete account of the exhuming of a mighty temple in the land of the Pharohs, for centuries buried from sight in the drifting sands that have swept under the sublime evidences of a bygone civilization.

---

THE death rate of the globe is computed to be 67 a minute, 96,480 a day, and 36,214,300 a year, and the birth rate, 70 a minute, 100,800 a day, and 36,772,009 a year.

---

### AT MIDNIGHT.

Tell me, glowing stars on high
Do I perish when I die?
Or shall I be ever I?

Will my spirit have re-birth
And regain the things of worth
When my dust returns to earth?

Ye, too, perish; ye, too, fall;
Flash a moment;—then the pall.
Is that typical of all?

Boundless depths of glowing spheres.
Changeless in the changing years,
Seem to negative our fears.

Yet your changeless is all change!
Fleeting, flying on, ye range
Through the vortex vast and strange.

Other creatures, other men,
Cling upon you, live—and then
Do they die and live again?

—N. H. DOLE, in *N. E. Magazine.*

